# Identification of Functional SNPs in *BARD1* Gene and *In Silico* Analysis of Damaging SNPs: Based on Data Procured from dbSNP Database

**DOI:** 10.1371/journal.pone.0043939

**Published:** 2012-10-09

**Authors:** Ali A. Alshatwi, Tarique N. Hasan, Naveed A. Syed, Gowhat Shafi, B. Leena Grace

**Affiliations:** 1 Molecular Cancer Biology Research Laboratory, Department of Food Science and Nutrition, King Saud University, Riyadh, Saudi Arabia; 2 Research and Development Center, Bharathiar University, Coimbatore, Tamil Nadu; 3 Department of Biotechnology, Vinayaka Missions University, Salem, Tami Nadu; Ohio State University Medical Center, United States of America

## Abstract

**Background:**

The *BARD1* gene encodes for the *BRCA1*-associated RING domain (BARD1) protein. Germ line and somatic mutations in *BARD1* are found in sporadic breast, ovarian and uterine cancers. There is a plethora of single nucleotide polymorphisms (SNPs) which may or may not be involved in the onset of female cancers. Hence, before planning a larger population study, it is advisable to sort out the possible functional SNPs. To accomplish this goal, data available in the dbSNP database and different computer programs can be used. To the best of our knowledge, until now there has been no such study on record for the *BARD1* gene. Therefore, this study was undertaken to find the functional nsSNPs in BARD1.

**Result:**

2.85% of all SNPs in the dbSNP database were present in the coding regions. SIFT predicted 11 out of 50 nsSNPs as not tolerable and PolyPhen assessed 27 out of 50 nsSNPs as damaging. FastSNP revealed that the rs58253676 SNP in the 3′ UTR may have splicing regulator and enhancer functions. In the 5′ UTR, rs17489363 and rs17426219 may alter the transcriptional binding site. The intronic region SNP rs67822872 may have a medium-high risk level. The protein structures 1JM7, 3C5R and 2NTE were predicted by PDBSum and shared 100% similarity with the BARD1 amino acid sequence. Among the predicted nsSNPs, rs4986841, rs111367604, rs13389423 and rs139785364 were identified as deleterious and damaging by the SIFT and PolyPhen programs. Additionally, I-Mutant showed a decrease in stability for these nsSNPs upon mutation. Finally, the ExPASy-PROSIT program revealed that the predicted deleterious mutations are contained in the ankyrin ring and BRCT domains.

**Conclusion:**

Using the available bioinformatics tools and the data present in the dbSNP database, the four nsSNPs, rs4986841, rs111367604, rs13389423 and rs139785364, were identified as deleterious, reducing the protein stability of BARD1. Hence, these SNPs can be used for the larger population-based studies of female cancers.

## Background

A single-nucleotide polymorphism (SNP) is the most common type of genetic mutation. There are several publically available databases for SNPs, such as dbSNP, GWAS Central, SwissVar etc. dbSNP is the most extensive among all the databases. By release of 135 hosting number of human SNPs reached more than 50 million, including 535,660 synonymous and 873,308 non-synonymous SNPs [1]. Only the non-synonymous SNPs (nsSNPs), also called as missense variants are particularly important as they result in to changes in the translated amino acid residue sequence. It is likely that nsSNPs play a major role in the functional diversity of coded proteins in human populations and have been linked with many diseases. nsSNPs may affect the protein function by reducing protein solubility or by destabilizing protein structure and they may affect gene regulation by altering transcription and translation all in ways that may not be identified by structure or phylogeny-based features [2], [3], [4], [5].

It is estimated that breast cancer may affect one out of every eight women at some point in her lifetime. Only 10% of women have a hereditary predisposition to breast cancer. Meanwhile, less than half of the patients have been found to carry a mutation in the *BRCA1* or *BRCA2* gene [6]. The disease may occur due to mutations in the code for the genes of the proteins that interact with BRCA1 and BRCA2. *BARD1* is one of these genes and encodes the BRCA1-associated RING domain protein (BARD1). BARD1 is a protein with 777 residues. It contains an amino-terminal RING domain (residues 46–90), three ankyrin repeats (residues 427–525) and two carboxy-terminal BRCT domains (residues 616–653 and 743–777). It also has nuclear export and localization signals (residues 102–120 and the residues after 177, potentially residues 204–209) [7]. BARD1 makes a stable heterodimer in association with BRCA1 [8]. Many mutations have been identified in *BARD1* in non-hereditary site-specific breast and breast/ovarian cancer cases [9], [10]. The majority of breast cancer cases (approximately 70%) are considered sporadic in nature because they do not have extensive familial history [11]. In most of these cases, *BRCA1* and *BRCA2* are rarely found mutated. In contrast, both germline and somatic *BARD1* mutations are found in sporadic breast, ovarian and uterine cancers [12].

A somatic mutation (Val695Leu) and a germline mutation in *BARD1* associated with sporadic breast cancer (Val695Leu) and one (Gln564His) associated with ovarian cancer have been reported [12]. Three SNPs namely, Lys312Asn, Cys557Ser and Asn295Ser have been found associated with *BRCA1* and *BRCA2* mutations in negative familial breast/ovarian cancer [9]. In spite of those findings, the functional role of *BARD1* in cancer susceptibility is unclear. However, many SNPs have been reported in *BARD1* but only two have been suggested to be involved into breast cancer susceptibility. Val507Met is considered to be responsible for high risk of postmenopausal breast cancer and Cys557Ser for familiar breast cancer [10], [13]. In addition of the female specific cancers, *BARD1* SNPs have been found to be associated with neuroblastoma. As a matter of fact, SNPs in *BARD1* coding region cause the expression of an oncogenic isoform and that influence the neuroblastoma susceptibility and oncogenicity [14] (Bosse et al, 2012). *BARD1* seems a plausible target for female-specific cancer and other cancer studies. However, knowledge about the clinical relevance for many of the *BARD1* SNPs is still limited [9], [10], [12]. This study was undertaken to explore and extend the knowledge related to the effect of SNPs on the stability and function of the *BARD1* gene.

## Results and Discussion

**Figure 1:**
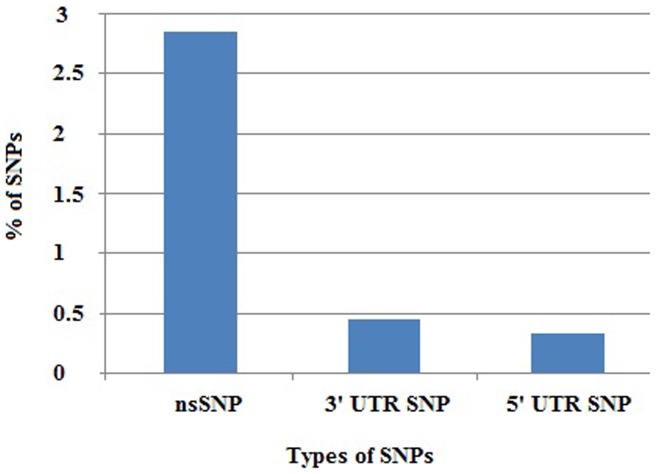
A graphical representation of distribution of nonsynonymous, 5′UTR and 3′ UTR SNPs for *BARD1* gene (based on the dbSNP database).

The dbSNP database contains both validated and non-validated polymorphisms. In spite of this drawback, we opted to avail the dbSNP because allelic frequency of most of nsSNPs of *BARD1* has been recorded there (except 12 out of 50) and that is the most extensive SNP database [15]. At dbSNP, *BARD1* gene contains data for 1709 SNPs. Out of 1752 SNPs, 50 are nsSNPs and 14 are in UTRs. There are 6 SNPs in the 5′ UTR and 8 SNPs in the 3′ UTR. Our investigation accounted for the nsSNPs in the coding region and the 5′ and 3′ UTR SNPs. A graphical representation of the distribution of SNPs in the coding region and the UTRs is depicted in terms of percentage in Figure 1; 2.85% of the total numbers of SNPs are nsSNPs present in the coding region, whereas only 0.34% and 0.45% of the total number of SNPs are in the 5′ and 3′ UTRs, respectively.

### Deleterious nsSNPs predicted by SIFT

The sequence homology-based tool SIFT was used to determine the level of conservation of a particular amino acid position in a protein. SIFT has been tested on many human SNP databases and was found able to distinguish the disease associated SNPs from a neutral one with only a 20% false positive error. The sensitivity of SIFT is confirmed by the subset of nsSNP from dbSNP predicted to affect function were involved in disease. Furthermore, The SIFT algorithm works mainly sequence for prediction while that performs similarly to tools that use structure. Since, SIFT can predict a large number of a substitutions, as that do not requires the structures. Seventy four percent (74%) of nsSNPs identified by the SNP Consortium, were sufficiently similar to homologs in protein sequence databases for SIFT prediction. Hence, using SIFT is advantageous over other tools [16].

**Figure 2:**
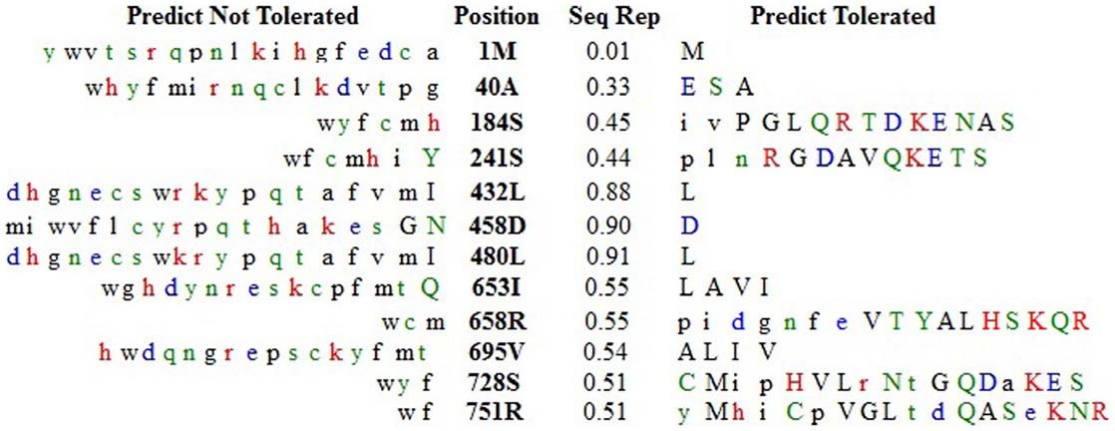
Sequence homology-based results from Sorting Intolerant from Tolerant (SIFT) server for SNPs (SIFT output is modified and depicted only the intolerant amino acid substitutions).

A .txt file containing “db SNP rsIDs” of all 50 nsSNPs was submitted to the “SIFT dbSNP rsIDs” page (http://sift.jcvi.org/www/SIFT_dbSNP.html) to calculate the tolerance index. The functional impact of the amino acid substitution is inversely proportional to the tolerance index (TI). Figure 2 and Table 1 summarize the results. Out of 50 nsSNPs 11 were predicted as ‘Not Tolerable’ (Table 1) and had a Tolerance Index (TI) ≤0.05. The corresponding amino acid substitutions of rs143914387, rs1048108, rs71579841, rs61754118 and rs139785364 had a TI score of 0.00. The TI score was 0.01 for rs187590361 and rs13389423, 0.02 for rs111367604, 0.03 for rs146629794 and 0.04 for rs3738885 and rs4986841. The nucleotide change C**→**T accounted for the maximum number (four) of deleterious SNPs, followed by A**→**G (two). The rest of the nucleotide changes occurred only once.

**Table 1:** List of nsSNPs predicted by SIFT as Not Tolerated.

SNP IDs	Nucleotide Change	Amino Acid Change	Tolerance Index
rs143914387	G/T	Q11H	0.00
rs1048108	C/T	P24S	0.00
rs71579841	C/T	A40V	0.00
rs3738885	C/G	S241C	0.04
rs146629794	C/A	A613E	0.03
rs4986841	A/T	I653F	0.04
rs187590361	A/G	N663S	0.01
rs111367604	G/C	V695L	0.02
rs13389423	C/T	S728F	0.01
rs61754118	A/G	I738V	0.00
rs139785364	C/T	R751W	0.00

### Damaging nsSNPs predicted by PolyPhen

The nsSNPs involved in structural modification were determined by the PolyPhen (Polymorphism and Phenotype) program. PolyPhen software version 2.0.9 predicts the fate of the structure and function of a protein due to an amino acid change through specific empirical rules on the sequence. Input options for the tool are protein sequence, accession number or database ID/accession number combined with sequence position with amino acid variants. For sequence-based characterization of the substitution site PolyPhen uses the TMHMM algorithm, Coils2 program and SignalP program to predict transmembrane, coiled coil and signal peptide regions of the protein sequences. PolyPhen identifies homologues of the input sequences via a BLAST and calculates position-specific independent count (PSIC) scores for every variant and estimates the difference between the variant scores, the difference of >0.339 is detrimental. The program carries out a BLAST query of a sequence against a protein structure database (PDB and PQS) for mapping of the substitution site to known protein 3-dimensional structures. PolyPhen uses the DSSP database to obtain secondary structure and solvent accessible surface area for the mapped amino acid residues. There are certain empirical rules applied on the sequences and the accuracy of that is approximately 82% with a chance of 8% false positive prediction [17].

The protein accession number of BARD1 (Q99728) and the amino acid substitutions corresponding to each of fifty nsSNPs were submitted separately. Table 2 summarizes the results obtained from the PolyPhen server. A position-specific independent count (PSIC) score difference was assigned using the categories ‘probably damaging’ (2.00 or more), ‘possibly damaging’ (1.40–1.90), ‘potentially damaging’ (1.20–1.50), ‘borderline’ (1.00–1.20) and ‘benign’ (0.00–0.90). Twenty-seven out of 50 nsSNPs were predicted as ‘damaging,’ and the PSIC scores fell into the range of 1.51 to 3.41. Five nsSNPs predicted to be deleterious by the SIFT program were also predicted to be ‘damaging’ by the PolyPhen server. rs139785364 had a SIFT TI of 0.00 and a PolyPhen PSIC of 2.495. Therefore, the relevant mutation would be important when manifesting itself in the cancers caused by the nonfunctioning of the BRCA1-BARD1 complex.

**Table 2:** List of nsSNPs that were predicted to be significantly damaging by PolyPhen.

SNP IDs	Nucleotide Change	Amino Acid Change	PSIC SD
rs140254589	A/G	D 102 N	1.614
rs144856889	C/T	H 116 Y	2.082
rs184660818	C/T	S 184 F	1.998
rs16852741	A/G	S 186 G	1.633
rs138593305	T/C	L 220 S	1.804
rs145009419	A/G	E 223 G	2.039
rs151325889	C/A	P 246 Q	2.087
rs138904906	A/G	N 255 S	1.736
rs146223579	T/C	I 258 T	1.568
rs148760338	C/T	P 315 L	2.299
rs141351035	T/G	C 362 G	2.902
rs2229571	G/C	R 378 S	1.79
rs76824305	T/G	V 422 G	1.527
rs137988817	G/C	D 458 H	2.064
rs111350417	T/C	V 477 A	1.51
rs149839922	T/C	L 480 S	1.885
rs146946984	G/A	R 565 H	1.859
rs75709313	C/A	A 594 D	1.863
rs140642433	T/C	C 628 R	3.41
rs4986841	A/T	I 653 F	1.718
rs187590361	A/G	N 663 S	1.669
rs111284953	T/A	V 695 D	1.879
rs150121935	A/T	D 710 V	2.514
rs140729292	G/A	A 721 T	1.268
rs13389423	C/T	S 728 F	1.998
rs76744638	C/G	R 731 G	2.27
rs139785364	C→T	R 751 W	2.495

### Functional SNPs in untranslated regions (UTR) predicted by FastSNP

The polymorphisms in the 3′ UTR affect gene expression during translation of mRNA while the polymorphisms in the 5′ UTR influence RNA half-life by altering polyadenylation [18], [19]. The FastSNP (Function Analysis and Selection Tool for Single Nucleotide Polymorphisms) program was used to predict the functionally important SNPs in the 3′ and 5′ UTRs. That is a web server that efficiently identifies the functional SNPs. That prioritizes SNPs according to twelve parameters (phenotypic risks and functional effects), such as changes to the transcriptional level, pre-mRNA splicing, protein structure, etc. FastSNP is unique in its feature that the prediction of functional effects is always based on the most up-to-date information. FastSNP extracts updated information from eleven external Web servers. FastSNP also provides project management services for registered users to store and export their candidate SNPs and update the SNPs putative functional effects by re-submitting the query [20].

The FastSNP search was performed by querying by gene symbol (*BARD1*). Table 3 lists the SNPs in the UTRs and the intronic region. The SNP rs58253676 in the 3′ UTR may have splicing regulator and enhancer functions and may possibly be a splice site. Most importantly, the nucleotide change may have a medium-high (3–4) level of risk for being a splicing regulator and a low-medium (2–3) level of risk for enhancer functions. rs17489363 and rs17426219 in the 5′ UTR may alter the transcriptional binding site. In the intronic region, rs67822872 SNP, an intronic enhancer, may have a medium-high (3–4) level of risk upon nucleotide change.

**Table 3:** List of SNPs intron and UTR (mRNA) predicted to be functionally significant by FastSNP.

SNP ID	Nucleotide Change	UTR/Intronic Position	Possible Functional Effect	Level of Risk
rs58253676	(>6 bp)	3′UTR	Splicing regulation	Very Low-Medium (2–3)
			Splicing Enhancer	Very Low-Medium (2–3)
			Splicing Site	Medium-High (3–4)
rs17489363	A/G	5′UTR	Promoter regulation	Very Low-Medium (2–3)
			Intronic Enhancer	Very Low-Medium (2–3)
			Change in transcription Binding Site	Yes
rs17426219	G/A	5′UTR	Promoter regulation	Very Low-Medium (2–3)
			Change in transcription Binding Site	Yes
rs67822872	–/A	Inton	Intronic enhancer	Medium-High (3–4)

### Modeling of amino acid substitution effects due to nsSNPs on protein structure, Energy minimization and RMSD

#### (A) The closest related protein structures

By using the EMBL-EBI Web-based tool PDBsum, the *BARD1* gene product-related protein structures were searched. Three related protein structures, namely 1JM7, 3C5R and 2NTE, were found to share 100% amino acid sequence similarity (Table 4). 1JM7 is a BRCA1-BARD1 complex. Chain B belongs to BARD1 and has 97 amino acid residues. Chain B accounts for residues 26 to 122. 3C5R and 2NTE are homodimers. They are the stretches of BARD1 that account for residues 425 to 545 and 568 to 777, respectively.

**Table 4:** The available PDB structure for the BARD1 gene with a similarity (100%) with BARD1 FASTA sequence at PDBsum.

PDB IDs	Length (AA)	Similarity	Chain used for study
1JM7	97	100%	Cain B
3C5R	125	100%	Chain A or B (Homodimer)
2NTE	210	100%	Chain A or B (Homodimer)

#### (B) Models of substituted amino acids and their minimized energy and RMSD

The single amino acid polymorphism database (SAAP) server http://www.bioinf.org.uk/saap/db/ is offline due to essential maintenance. Thus, we were unable to map the deleterious nsSNPs into protein structure through SAAP. 1JM7, 3C5R and 2NTE were scanned manually to identify amino acid polymorphisms. IJM7 accounted for three nsSNPs: rs71579841 (Ala40Val), rs140254589 (Asp102Asn) and rs144856889 (His116Tyr). 3C5R also had three nsSNPs: rs137988817 (Asp458His), rs111350417 (Val477Ala) and rs149839922 (Leu480Ser). However, 2NTE had 10 nsSNPs. nsSNPs found in 1JM7, 3C5R and 2NTE are listed in Table 5. All the functional nsSNPs predicted using the SIFT and PolyPhan tools and present in the three structures mentioned above were subjected to the SPDBV mutation tool. A model for each functional nsSNP was made and visualized as a comparison using SPDBV.

**Table 5:** RMSD and total energy after energy minimization of native-structures of 1JM7, 3C5R and 2NTE and their mutant models.

Molecules	RMSD (Å)	Total energy after energy minimization (KJ/mol)
*1JM7 native-type structure*		*−5209.592*
1JM7 Mutant 40 (rs71579841 )	0.1364	−2218.149
1JM7 mutant 102 (rs140254589)	0.7866	−5389.294
1JM7 mutant 116 (rs144856889)	1.8039	−5464.582
*3C5R native-type structure*		*−6174.53*
3C5R mutant 458 (rs137988817)	1.1598	−6126.259
3C5R mutant 477 (rs111350417)	0.1626	−6166.61
3C5R mutant 480 (rs149839922)	0.5496	−6126.997
*2NTE native-type structure*		*−12127.86*
2NTE mutant 594 (rs75709313)	0.2848	−12146.148
2NTE mutant 628 (rs140642433)	0.6725	−11952.286
2NTE mutant 653 (rs4986841)	0.2891	−12192.765
2NTE mutant 663 (rs187590361)	0.3793	−12020.879
2NTE mutant 695 (rs111367604)	0.4299	−12263.969
2NTE mutant 710 (rs150121935)	0.5399	−12018.463
2NTE mutant 721 (rsrs140729292)	0.696	−12200.248
2NTE mutant 728 (rs13389423)	0.4079	−12134.148
2NTE mutant 731 (rs76744638)	0.4724	−11862.29
2NTE mutant 751 (rs139785364)	0.1796	−11881.313

Energy minimization for all the models and their native structures was achieved using the NOMAD-REF Gromacs server. The Gromacs tool uses a force field for energy minimization. The total energy for all the mutant and native models after minimization is listed in Table 5. The total energies for the native structures of 1JM7, 3C5R and 2NTE are −5209.592 kJ/mol, −6174.53 kJ/mol and −12127.86 kJ/mol, respectively. Change in total energy due to mutation is noticeable in the 1JM7 mutant rs71579841 (Ala40Val), being −2218.149 kJ/mol. Change in the total energy due to mutation is also noticeable in the 2NTE mutants rs76744638 (Arg731Gly) and rs139785364 (Arg751Try), being −11862.29 kJ/mol and −11881.313 kJ/mol, respectively. Interestingly, other mutant models had almost the same energy as their native structures.

RMSD is the measure of the deviation of the mutant structures from their native configurations. Higher the RMSD value, the more deviation between the two structures. Structure changes, in turn, affect functional activity. Among all the 16 mutants, rs144856889 (His116Tyr) had the highest RMSD (1.8039 Å), followed by rs137988817 (Asp458His) (1.1598 Å). rs140254589 (Asp102Asn), rs140729292 (Ala721Thr), rs140642433 (Cys628Arg), rs149839922 (Leu480Ser) and rs150121935 (Asp710Val) had 0.6960 Å, 0.6725 Å, 0.5496 Å and 0.5399 Å RMSD scores, respectively. Scores for other mutants fall in the range between 0.1364 (rs71579841, Ala695 Val) and 0.4724 (rs76744638, Arg731Gly). RMSDs for all the mutant structures are listed in Table 5.

### Prediction of change in stability due to mutation

The I-Mutant 2.0 server was used to predict the change in protein structure stability due to mutations. the input option for this tool is the 3D structure of protein. The tool was developed and tested with the data extracted from ProTherm which is the most comprehensive available database of thermodynamic experimental data of free energy changes of protein stability due to mutation. Hence, that efficiently predicts whether a protein mutation affects the stability of the protein structure or not. The predictions are 80% or 70% accurate depending upon the usage of structural or sequence information, respectively. The tool provides the scores of free energy change predictions calculated with the energy-based FOLD-X tool. By incorporating the FOLD-X approximation with those of I-Mutant, an precision of 93% on one third of the database can be accomplished, thus making I-Mutant a helpful tool for protein design and mutation [21].

Although the stabilities of the two nsSNPs rs144856889 (His116Tyr) and rs137988817 (Asp458His) increased, their reliability index (RI) was zero (0) and one (1), respectively. Other mutants exhibited decreased stability with an RI ranging between 9 and 3. These results are summarized in Table 6.

**Table 6:** Predictions of protein stability change due to mutations.

Molecules Models	Position of amino acid on protein Molecule	WT	MT	SVM2 Prediction Effect	DDG Value Prediction Kcal/mol	RI	RSA
**1JM7 Mutant Models**							
1JM7 (rs71579841)	40	A	V	Decrease	−0.53	3	0.0
1JM7 (rs140254589)	102	D	N	Decrease	−2.33	9	20.8
1JM7 (rs144856889)	116	H	Y	Increases	−0.13	0	74.2
**3C5R Mutant Models**							
3C5R (rs137988817)	458	D	H	Increases	−0.26	1	8.3
3C5R (rs111350417)	477	V	A	Decreases	−1.29	8	0.0
3C5R (rs149839922)	480	L	S	Decreases	−2.15	6	0.0
**2NTE Mutant Models**							
2NTE (rs75709313)	594	A	D	Decrease	−1.25	6	13.6
2NTE (rs140642433)	628	C	R	Decrease	−1.84	7	4.3
2NTE (rs4986841)	653	I	F	Decrease	−0.51	8	3.8
2NTE (rs187590361)	663	N	S	Decrease	−1.49	6	10.2
2NTE (rs111367604)	695	V	L	Decrease	−0.7	8	0.7
2NTE (rs150121935)	710	D	V	Decrease	−1.07	4	59.0
2NTE (rsrs140729292)	721	A	T	Decrease	−1.02	6	0.0
2NTE (rs13389423)	728	S	F	Decrease	−0.41	3	6.8
2NTE (rs76744638)	731	R	G	Decrease	−0.88	6	31.4
2NTE (rs139785364)	751	R	W	Decrease	0.15	7	37.6

For all the predictions, pH and temperature were selected as 7.0 and 25°C, respectively. WT: Wild type amino acid, MT Mutant type amino acid, ΔΔG: ΔG(New Protein)-ΔG(Wild Type) in Kcal/mol (ΔΔG<0: Decrease stability, ΔΔG>0: Increase stability), RI: Reliability index, RSA: Relative solvent accessible area.

### Mutant amino acids affect the domain structures of BARD1

**Figure 3:**
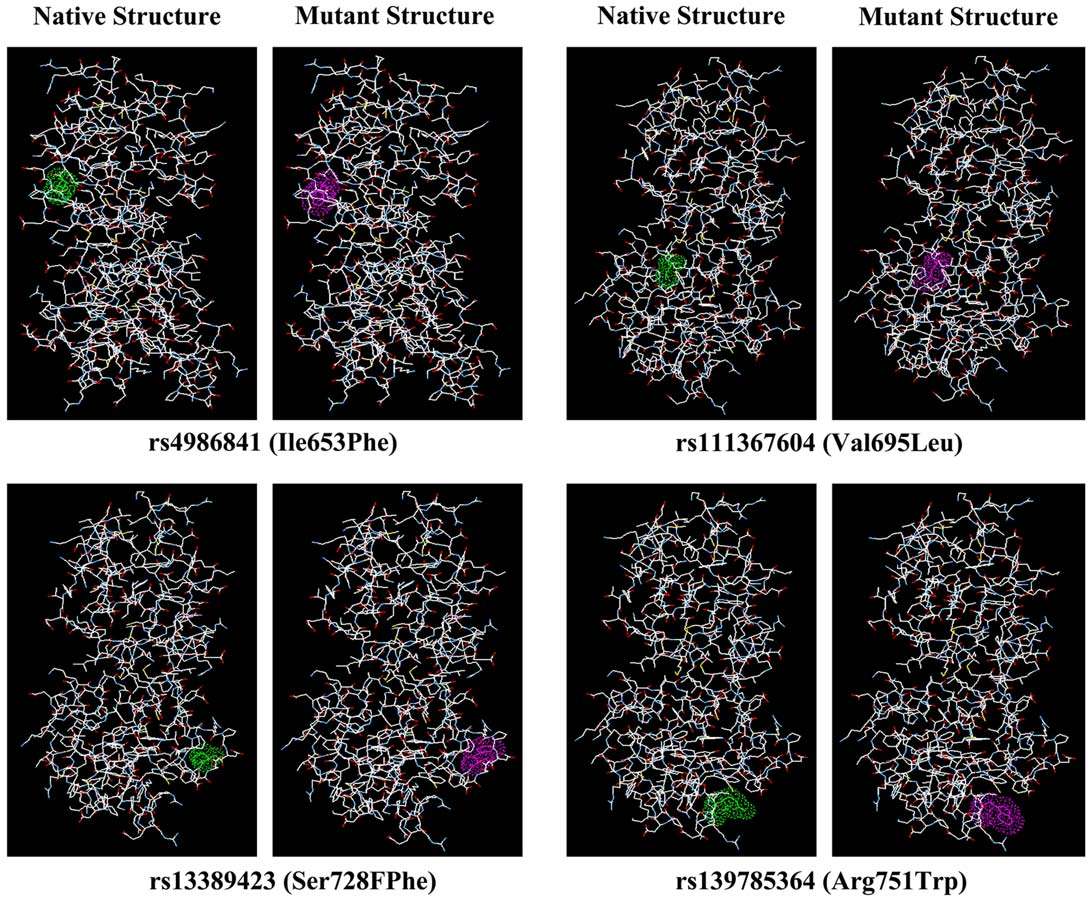
A comparison of amino acid substitutions due to nsSNPs, rs4986841 (Ile653Phe), rs111367604 (Val695Leu), rs13389423 (Ser728FPhe) and rs139785364 (Arg751Trp). Figure shows the differences of structure and electron cloud density between native and mutant models BARD1 Protein (PDB ID: 2NTE). Models were generated by using SPDBV (v4.0).

The affected domains and the allelic frequency of corresponding nsSNPs are listed in Table 7. Only ankyrin ring and BRCT domains harbor the predicted deleterious mutations. All the mutations of 2NTE, except rs187590361 (Asn663Ser), were located in the BRCT domains of BARD1, whereas all the 35CR mutations were located in the ankyrin rings. Structural changes in BARD1 due to 2NTE mutations can be better understood in Figure 3.

**Table 7:** ns SNPs found in different motifs and domains of BARD1 protein.

Structural domains	Mutants	Amino Acid Position	Wild Type	Mutant	Allelic Frequency
**Ankyrin Repeat**	3C5R (rs137988817)	458	D	H	0.00
	3C5R (rs111350417)	477	V	A	0.5
	3C5R (rs149839922)	480	L	S	NA
**BRCT domain**	2NTE (rs75709313)	594	A	D	0.05
	2NTE (rs140642433)	628	C	R	0.0
	2NTE (rs4986841)	653	I	F	0.01
	2NTE (rs111367604)	695	V	L	0.05
	2NTE (rs150121935)	710	D	V	0.0
	2NTE (rsrs140729292)	721	A	T	0.0
	2NTE (rs13389423)	728	S	F	0.019
	2NTE (rs76744638)	731	R	G	0.5

One of the major purposes of genetics studies is to distinguish functionally neutral mutations from those that contribute to disease. About half of the known gene lesions accounting for human inherited disease involve amino acid substitutions. Hence, to identify the nsSNPs those affect protein functions and, in turn, manifest themselves as diseases are an important issue [22], [23]. The functional effect of many nsSNPs may be neutral because natural selection will have removed mutations in essential positions. Using phylogenetic information with certain structural approaches is the basis of the assessment of these nsSNPs. Still, there is increasing evidence that the onset of many human diseases is due to mutations in the intronic regions of genes. Such mutations cause alterations in regulatory regions and the splicing process [24], [25].

SNPs are widespread throughout the genome. This fact makes them a preferred choice as genetic markers in the research on diseases and their corresponding drugs [26]. More than 1 million SNPs have been reported so far. Many of them provide a large amount of information about relationships between individuals, populations and diseases. However, the large numbers of SNPs cause a challenge for biologists and bioinformaticians [26]. Studying associations between disease risk and these genetic variations using a molecular epidemiological approach has gained much attention from scientists. The number of reported and recorded SNPs is increasing. This huge number of SNPs makes it difficult for researchers to plan costly population-based genotyping. Due to a plethora of SNPs, it is difficult to choose the target SNPs that will most likely affect phenotypic functions and ultimately contribute to disease development [24], [26], [27].

Approximately 5–10% of breast and ovarian cancer predispositions are hereditary [28] *BRCA1* and *BRCA2* being the most studied susceptibility genes. Mutations in *BRCA1* are found in 40–50% of families with a high breast cancer risk. Among these mutation occurrences, 75–80% account for both breast and ovarian cancers [29]. Even so, a significant proportion of predisposition to breast cancer that is due to these genetic aberrations is still unanswered. This leads us to hypothesize that there must be involvement of some other susceptibility genes. Therefore, we targeted the genes encoding proteins associated to BRCA1 for study.

In this study, we have examined the *BARD1* gene to analyze and identify the deleterious and functional nsSNPs using *in silico* methods. BARD1 is one of the BRCA1-associated proteins and the two share closely related domain structures [30]. Both have an N-terminal zinc finger domain and a C-terminal BRCT domain which had been found in many proteins. In these proteins, the domains are involved in DNA repair and cell cycle regulation. Particularly, the zinc finger domain is functionally important in the formation of the BRC1/BARD1 complex [31]. BARD1 contains three ankyrin repeats, which have been reported to be involved in transcription regulation when they are also present in other proteins [32]. Furthermore, the complex of the BARD1/BRCA1 heterodimer and CstF-50 (cleavage stimulation factor subunit 1) represses the polyadenylation machinery, presumably to prevent inappropriate mRNA processing at sites of DNA repair [33]. BARD1 also regulates the nuclear translocation of BRCA1 by preventing its export [34]. The involvement of BARD1 in TP53-independent apoptotic signaling has been reported previously. It can also function independent of BRCA1. BARD1 interacts with ankyrin repeats of BCL3 and thus is likely to modulate the activities of the transcription factor NFKB [35], [36].

Hence, nsSNP variation which causes a change in amino acid composition may result in the alteration of structural domains. For example, if there is an alteration in the ring finger domain, it may hinder BRCA1/BARD1 complex formation, reduce the stability of BRCA1, and change the polyadenylation process of mRNAs. Nevertheless, the alteration of ankyrin repeats or the BRCA1 C-terminal (BRCT) domain may lead to abnormal transcriptional or cell cycle regulation, respectively.

SIFT predicted 11 nsSNPs as deleterious, and PolyPhen predicted 27 nsSNPs as deleterious. Among them, only 5 nsSNPs were common (Table 1 and 2). They are rs4986841 (Ile653Phe), rs187590361 (Asn663Ser), rs111367604 (Val695Leu), rs13389423 (Ser728Phe) and rs139785364 (Arg751Trp). rs111367604 (Val695Leu) has been found to be associated with predisposition to breast, ovarian and uterine cancers [12], which is in agreement with our findings. SNPs in UTRs may alter transcription binding sites, splicing sites and polyadenylation of mRNAs [18], [19]. The SNPs rs58253676 in the 3′ and rs17489363 and rs17426219 in the 5′ UTRs are predicted to be involved in splice site regulation (Table 3). None of them have been studied so far in terms of their functional effects in any population. There were only three structures found in the Protein Data Bank, 1JM7, 3C5R and 2NTE, which shared 100% similarity with the BARD1 amino acid sequence (Table 4). Energy minimization, RMSD calculation and modeling of mutants were performed on the above-mentioned structures. The free energies of the mutant models of 1JM7 rs71579841 (Ala40Val), C35R rs137988817 (Asp458His), C35R rs149839922 (Leu480Ser) and 2NTE rs140642433 (Cys628Arg) and rs76744638 (Arg731Gly) decreased markedly. The minimum RMSD was calculated to be 0.1364 for 1JM7 rs71579841 (Ala40Val), while the maximum RMSD was calculated to be 1.8039 for 1JM7 rs144856889 (His116Tyr) and 1.1598 for C35R rs137988817 (Asp458His). RMSDs in the range of 0.7866 for rs140254589 (Asp102Asn) and 0.1796 for rs139785364 (Arg751Trp) (Table 5) were observed in these mutants. All five nsSNPs which were predicted to be deleterious by both SIFT and PolyPhen were found to be involved in decreasing protein stability. Four of them, rs4986841 (Ile653Phe), rs111367604 (Val695Leu), rs13389423 (Ser728Phe) and rs139785364 (Arg721Trp), were found in the BRCT domain of BARD1. This finding suggests that these four nsSNPs may decrease protein stability, hinder transcriptional regulation, and interfere with cell cycle regulation [31].

BARD1 SNPs G1743C (Cys557Ser), T2006C (Cys645Arg) and G2355A (Ser761Asn) have been identified to be associated with ovarian cancer, breast and ovarian cancer and breast cancer, respectively [9], [12]. A Finnish population study reported three synonymous and four nsSNPs. The nsSNPs were C1207G (Ser378Arg), G1592A (Val507Mat), C2045T (Arg658Cys) and G1743C (Cys557Ser). Only G1743C (Cys557Ser) was found associated with breast cancer predisposition in that study [10]. The same SNP was found associated with risk of single and multiple primary breast cancer [37]. Pro24Ser and C1207G (Ser378Arg) may jointly contribute to the susceptibility of breast cancer. Their heterozygote and homozygote are associated with decreased risk of breast cancer [38]. Recently, G1743C (Cys557Ser) has been reported for no association with the predisposition of familial breast cancer in an Australian population based case control study [39]. In a cohort based study of a French population, nine common SNPs of *BARD1* including G1743C (Cys557Ser) were not shown any role as modifier of risk in *BRCA1/2* mutant carriers [40]. Furthermore, *BARD1* SNPs rs6435862 and rs3768716 and some known common SNPs has been found significantly associated with the aggressive neuroblastoma [41].During the last decade, approximately 12 nsSNPs have been studied in different populations for their association with the predisposition to various female cancers. Some of them are recorded in the dbSNP database for the *BARD1* gene (http://www.ncbi.nlm.nih.gov/gene/580). They are rs28997576 (Cys557Ser), rs146946984 (Arg565His), rs34744268 (Cys645Arg), rs111367604 (Val695Leu) and rs142155101 (Ser761Asn) [9], [12]. rs111367604 (Val695Leu) has been predicted to be deleterious by SIFT and PolyPhen, while I-mutant also predicts its decreased stability. rs146946984 (Arg565His) has been predicted to be deleterious by PolyPhen only. Contrarily, among nsSNPs predicted damaging by SIFT and/or PolyPhen rs1048108 (Ser24Pro), rs16852741 (Gly186Ser) rs2229571 (Ser378Arg) had been reported in population based studies. Studies revealed that above three nsSNPs show no significant association with disease [42], [43]. Although, rs28997576 (Cys557Ser), rs34744268 (Cys645Arg) and rs142155101 (Ser761Asn) are well studied and published nsSNPs, however, none of the tools used for the predictions were able to predict their damaging effects. Hence, there is a need of testing the predicted nsSNPs for their functional roles meanwhile; there is also a need of improveing the web-based tools for more précised predictions. Many nsSNPs have been studied in populations but not indexed in the dbSNP database, such as Asn295Ser, Lys312Asn, Asn470Ser, Gln564His, Thr598Ile and Ile692Thr [9], [12], [13], [44]. Thus, there is also a need to update the dbSNP database accordingly.

## Conclusions

This study concludes that with the available bioinformatics tools and the data present in the dbSNP database, four snSNPs are deleterious and likely reduce protein stability. These snSNPs are rs4986841 (Ile653Phe), rs111367604 (Val695Leu), rs13389423 (Ser728FPhe) and rs139785364 (Arg751Trp). Their presence in the BRAC domain increases the possibility of altered transcriptional and cell cycle regulation. Therefore, the probability of their involvement in disease predisposition increases. This prediction can be further tested through larger population-based studies.

## Materials and Methods

### Datasets

*BARD1* gene SNPs and their protein sequences in the FASTA format were retrieved from the dbSNP database [15], [45] (http://www.ncbi.nlm.nih.gov/SNP/), and “.pdb” files for BARD1 subunits were retrieved from the RCSB Protein Data Base [46] (http://www.rcsb.org/pdb/home/home.do) for computational analysis in this study.

### Sequence homology-based prediction of deleterious nsSNPs by using SIFT

The Sorting Intolerant from Tolerant (SIFT) server available at (http://sift.jcvi.org) was used to predict the deleterious coding non-synonymous SNPs. The SIFT program can sort out the functionally neutral and deleterious amino acid changes due to SNPs in the coding regions of genes [16]. For the prediction of functional consequences on proteins due to nsSNPs, the SIFT program utilizes amino acid sequence homology and the physical properties of the proteins in combination with naturally occurring nsSNPs by aligning paralogous and orthologous protein sequences. The algorithms for the SIFT program use the latest SWISS-PROT, nr and TrEMBL databases to find homologous sequences by considering the median conservation sequence score to be 3.00. The threshold for the intolerance index is ≥0.05. Seq-Rep is the fraction of sequences that contain amino acids shown in color code: black (non-polar); green (uncharged polar); red (basic); blue (acidic). A low fraction indicates the position is either severely gapped or non-alignable and has little information.

### Structural homology-based prediction of functional consequences of coding nsSNPs by using PolyPhen

The ability of the protein to interact with other molecules or to have different functions depends upon its tertiary structure [47], [48]. Therefore, analysis of damaged coding nsSNPs at the structural level is necessary to understand the activity of the protein. The Polyphen server (http://genetics.bwh.harvard.edu/pph/) was used to study the functional consequences of nsSNPs [17], [49]. The PolyPhen server requires the protein sequence or a SWALL database ID or accession number as well as the sequence position of amino acid variants. PolyPhen classifies the SNPs as “benign,” “possibly damaging” or “probably damaging” based on site-specific sequence conservation among mammals, as well as their location in the three-dimensional structure of the protein molecule. The term “damaging” used by PolyPhen reflects the mutations affecting protein structure and not the loss or gain of function [50]. The protein identifier from the UniProt database for the BARD1 protein “Q99728” was submitted with the position of variation along with the wild type and mutant amino acids. PolyPhen then calculated PSIC scores for each of the two variants based on three parameters, namely, (i) sequence-based characterization of the substitution site, (ii) profile analysis of homologous sequences and (iii) mapping of a substitution site to a known three-dimensional protein structure. The PSIC score difference between the two variants elucidates the amount of functional consequences that the nsSNP exerts. The PSIC score difference is regarded to be directly proportional to the impact of a particular amino acid substitution [51].

### Scanning of functional SNPs in untranslated regions (UTRs) of the BARD1 gene using FastSNP

SNPs in the UTR sites are involved in the regulation of gene expression in many ways, such as RNA transcript splicing site or transcription factor binding site alteration [52], [53]. Hence, the UTRs were also analyzed for their functional SNPs. FastSNP (http://fastsnp.ibms.sinica.edu.tw) prioritizes SNPs according to twelve phenotypic risks and putative functional effects, such as changes to the transcriptional levels and pre-mRNA splicing and protein structure. The input order for the candidate SNPs was (i) input the candidate gene using the gene symbol, (ii) input a single SNP “rsID” or a list of SNP rsIDs for batch analysis and (iii) paste the novel SNP sequence. Input of the candidate gene symbol (BARD1) was used for analysis. Finally, the 3′and 5′ UTRs were analyzed. The SNP prioritization result was a list of SNPs with its risk ranking and possible function types. Risk level is ranked as 0, 1, 2, 3, 4 or 5, which signify the levels of “no risk”, “very low risk,” “low risk,” “medium risk,” “high risk,” and “very high risk,” respectively.

### Modeling of protein structure amino acid substitutions caused by nsSNPs, energy minimization and calculating the RMSD

#### (A) Finding the closest related protein

The EMBL-EBI Web-based tool PDBsum (http://www.ebi.ac.uk/pdbsum/) was used to find the proteins related to the BARD1 gene. PDBsum provides an at-a-glance overview of every macromolecular structure deposited in the Protein Data Bank (PDB). It performs a FASTA search against all sequences in the protein data bank (PDB) to obtain a list of the closest matches. The FASTA sequence of the BARD1 protein was provided in the query space. We selected only the three closest matches, namely the solution structure of the BRCA1/BARD1 ring-domain heterodimer (PDB ID 1JM7) [8], the crystal structure of the BARD1 ankyrin repeat domain (PDB ID 3C5R) [54] and the crystal structures of the BARD1 BRCT domains (PDB ID 2NTE) [55].

#### (B) Modeling amino acid substitution, energy minimization and RMSD calculation

Swiss-PDBViewer (v4.04) was used to generate the mutated models of each of the selected PDB entries for the corresponding amino acid substitutions. Swiss-PDBViewer allows browsing through a rotamer library to change amino acids. A “mutation tool” was used to replace the native amino acid with a new one. The mutation tool facilitates the replacement of the native amino acid by the “best” rotamer of the new amino acid. The “.pdb” files were saved for all the models. The NOMAD-Ref Gromacs server was used to perform energy minimization for all the native and mutated models of 1JM7, 3C5R and 2NTE. The NOMAD-Ref Server makes use of Gromacs using force fields for energy minimization according to the steepest descent, conjugate gradient or L-BFGS methods [56]. The conjugate gradient method was utilized in this study. RMSDs between the native structure and each mutant were calculated using YASARA [57].

### Predicting the change in stability due to mutation

To predict the change in the stability of the protein upon mutation, a support vector machine (SVM)-based tool server, I-Mutant 2.0, was used. This tool automatically predicts protein stability changes upon single point mutations. Prediction can be performed using either protein structure or sequence. I-Mutant 2.0 can be used both as a classifier for predicting the sign of the protein stability change upon mutation and as a regression estimator for predicting the related change in Gibbs-free energy (ΔΔG) [21]. *Scanning of nsSNPs for their position in different protein domains*

To find the nsSNPs and the amino acid changes they may cause in different domains of the protein structures, the Prosit-ExPaSy tool was used (http://prosite.expasy.org/). The UniProtKB ID was provided for the query column, and the UniProt database was searched for motifs and domains of BARD1. The results were obtained as the categorized sequence of amino acids with their respective positions in the protein subsequences and domains.
